# Evaluating National Health Systems: The Case of the General Health System of Cyprus Through a Survey

**DOI:** 10.1002/hsr2.71539

**Published:** 2026-01-06

**Authors:** George Evripides, Paul Christodoulides

**Affiliations:** ^1^ Department of Electrical Engineering, Computer Engineering and Informatics Cyprus University of Technology Limassol Cyprus; ^2^ Faculty of Engineering and Technology Cyprus University of Technology Limassol Cyprus

**Keywords:** descriptive statistics, healthcare, medical health system, questionnaire, satisfaction with MHS

## Abstract

**Background and Aims:**

A medical health system (MHS) comprises organizations, professionals, and resources working together to maintain and improve health at both individual and societal levels. Evaluating MHS performance is essential to ensure quality, equity, and sustainability. This study attempts an initial assessment of Cypriots' views on the newly developed General Health System (GHS) of Cyprus, a national MHS.

**Methods:**

First, a literature‐based review was performed on national MHSs in relation to their features and performance. Next, the GHS schedule and implementation were discussed. The literature review informed the design of a structured questionnaire, covering six constructs: satisfaction, trust, reliability, expectations, improvement factors, and comparison of the MHS before and after GHS implementation. A total of 445 responses were collected through stratified sampling across Cyprus. Descriptive statistics (means, standard deviations) and reliability analysis (Cronbach's *α*) were conducted.

**Results:**

Key measures such as Satisfaction (Mean [*M*] = 2.589), Trust (*M* = 2.406), and Reliability (*M* = 2.328) were below neutral (*M* = 3.0), suggesting negative perceptions. By contrast, Expectations were high (*M* = 4.256), indicating that citizens anticipate significant improvements from GHS. No significant variation was observed across gender, age, district, income, or work sector. Cronbach's *α* values confirmed strong internal consistency (0.80–0.93). Findings indicate moderately low levels of satisfaction, trust, and reliability in the GHS despite high public expectations.

**Conclusion:**

These results are consistent with prior national MHS surveys and highlight systemic rather than subgroup‐specific challenges. This study contributes the first nationwide assessment of the Cyprus GHS, offering evidence‐based insights for healthcare policy and service improvement.

## Summary

- Main aspects for a successful medical health system (MHS).
- Comparison of MHSs.
- Evaluation of the MHS of Cyprus through a questionnaire.
- Discussing the results in conjunction with already implemented MHS.

## 1 Introduction

A medical health system (MHS) comprises organizations, professionals, and resources working together to maintain and improve health at both individual and societal levels. Evaluating MHS performance is critical for globally ensuring equitable and effective healthcare delivery [1, 2].

A strong MHS is crucial for individuals. Table 1 lists the main criteria for a successful MHS [1, 3, 4]. A well‐functioning MHS not only protects individual health but also supports public health and socioeconomic development, making its evaluation essential. It underpins any society's attempts to promote healthy, productive lives. Its mission is to provide complete healthcare to individuals and communities, including prevention, diagnosis, treatment, and rehabilitation. Healthcare institutions with superior infrastructure and resources support important medical care in varied situations. Public and private financing enable universal care. Government oversight and regulation ensure system quality, safety, and equity.

**Table 1:** Main aspects of MHS.

Aspect	Comment
Access to healthcare services	Equitable service availability regardless of income, location, or health status.
Healthcare quality	Safe, effective, and patient‐centered care following standards.
Disease prevention and health promotion	Vaccination, screening, health education, and risk reduction.
Emergency and trauma care	Timely trauma and crisis response to reduce mortality.
Financial protection	Affordable care with reduced out‐of‐pocket expenses.
Health equity and social justice	Reduced disparities; focus on vulnerable populations.
Economic development	Healthy population supports productivity and growth.

Regarding MHSs' strengthening, this is often defined ambiguously, making monitoring and evaluation challenging. To this end, Bertone et al. [5] proposed 22 MHS process goals as tools for planning and evaluation. These highlight the need for citizens, governments, and stakeholders to critically assess approaches and the role of external support in building long‐term MHS capacity.

In another study, Ciulla et al. [6] compare European managed entry agreements (MEA) to US deregulated pharmaceutical pricing and their pros and cons. Despite low cost‐effectiveness, MEA have helped pharmaceutical businesses and national MHS access innovative medicines. The diversity and complexity of MEA, caused by expenditure management policy, recommend that standard regulations on efficacy, budget impact, price, reimbursement, and accessibility must be improved. These policy mechanisms illustrate how financing arrangements directly influence system performance and access to innovation, a challenge also relevant to Cyprus as it seeks to optimize its GHS.

Bogale et al. [7] reviewed MHS strengthening in fragile and conflict‐affected states, identifying governance, workforce capacity, and financing as essential foundations. Despite some progress, efforts were often undermined by insecurity, migration of health professionals, poor working conditions, and limited opportunities for training. HSS policies that showed promise included reintegration of displaced healthcare professionals, capacity‐building for local staff, use of supportive supervision, and adoption of e‐Health technologies.

Across OECD countries, health coverage is organized under three main models [8]. The first is the tax‐funded universal system, found in countries such as the UK, Denmark, and the Nordic states, where healthcare is primarily financed through general taxation and ensures broad public access. The second model is mandatory social health insurance, as in Germany, France, and Austria, where contributions from employers and employees are pooled to fund care, often supplemented by general revenues. The third model is a mixed or regulated private insurance system, seen in the Netherlands and Switzerland, where all citizens must purchase health insurance from competing providers, but government regulation ensures equity through subsidies and risk‐pooling mechanisms. While each model differs in financing and organization, all aim to balance access, quality, and sustainability [8].

In South Africa [9], the performance of the MHS continues to be shaped by deep historical inequalities and governance challenges. Despite reforms aimed at building a comprehensive and integrated public health service, persistent socioeconomic disparities, weak leadership, and policy implementation gaps have limited progress. Addressing these systemic barriers remains essential for improving equity and overall system performance.

Kaplan [10] proposed an evaluation framework to address the political, organizational, and social challenges often encountered in health informatics projects. The framework highlights four key dimensions—care, communication, context, and control—that shape the success or failure of medical information systems. By applying these dimensions, evaluators can better understand how organizational and social dynamics influence technological adoption and system performance.

Edward et al. [11] applied a Balanced Scorecard (BSC) to evaluate the Afghan MHS between 2004 and 2008, covering domains such as patient satisfaction, provider satisfaction, service capacity, and service quality. Over this period, measurable improvements were observed across all domains, including higher satisfaction levels, expanded service capacity, and better alignment with pro‐poor and pro‐female health policies. The study demonstrated that the BSC can serve as a practical monitoring and benchmarking tool, adaptable to changing healthcare priorities and policy frameworks.

Brazil's Unified Health System (UHS) [12] has significantly expanded healthcare access over the past four decades, driven largely by civil society rather than political elites. Despite these achievements, the system continues to face major challenges in financing, decentralization, and ensuring equity across regions with vast social and geographical disparities. Balancing the roles of the public and private sectors remains a central difficulty for sustaining universal coverage.

Indonesia's Jaminan Kesehatan Nasional (JKN) was launched with the ambitious goal of achieving universal health coverage by 2019, making it one of the largest single‐payer systems in the world [13]. While significant progress was achieved, around one‐third of the population remained uninsured, and even enrolled members often faced out‐of‐pocket payments for care. These challenges highlight the persistent difficulty of reaching vulnerable populations and ensuring financial protection, despite ongoing policy reforms and system integration efforts.

Post‐COVID, public satisfaction with MHS varied considerably across Europe. Countries with strong investment and well‐organized access, such as the Netherlands and Denmark, maintained relatively high levels of satisfaction due to efficient primary care and reduced financial barriers. By contrast, nations with lower health spending, including Greece and Portugal, faced greater challenges, with longer waiting times, workforce shortages, and unmet healthcare needs becoming more prominent [14, 15].

Public satisfaction is recognized by the World Health Organization (WHO) as an important indicator of MHS quality [16]. Although not a complete measure, it provides valuable insights into how healthcare systems are perceived and is increasingly used by policymakers to assess equity and responsiveness [17, 18]. Evidence shows that satisfaction levels are influenced by demographic and socioeconomic factors, with disadvantaged groups often experiencing more difficulty navigating healthcare systems [19, 20]. Limited health resources, lower health literacy, and unequal interactions with providers can further reduce satisfaction among low‐income and less‐educated populations [21, 22, 23]. These findings highlight the importance of considering both systemic investment and social inequalities when evaluating MHS performance.

In the current paper, the first systematic evaluation of the Cyprus GHS is conducted, focusing on its strengths, limitations, and areas for improvement through a structured questionnaire. Given that the system was only recently implemented, evidence on its performance and public perception remains limited. This study, therefore, provides timely insights into how citizens experience the new framework and highlights key areas where policy adjustments may be necessary to ensure its long‐term sustainability.

The rest of the paper is organized as follows. In Section Methods, 2, the methods used for achieving the goals of the current paper are presented, with main aspects of already established NHS addressed, with information about the GHS given, and with details on the construction of the questionnaire. In Section Results, 3, the statistical analysis of the questionnaire is presented. Finally, a discussion of the results (Section Conclusion, 4) and conclusions (Section 5) is given.

## 2 Methods

The first step in the present analysis was to review national MHSs to understand how they operate, how they compare, and which features are most salient.

For a better understanding of the GHS, it is useful for one to have in mind facts regarding national MHSs in various countries of the world, and in particular European Union (EU) countries, as Cyprus is a EU member state. Due to disparities in healthcare delivery structures, financial mechanisms, cultural contexts, and socioeconomic considerations, comparing national MHSs internationally is difficult. Due to the complexity and diversity of healthcare systems worldwide, comparing country's national MHSs with numbers and diagrams would be difficult. However, one can outline Healthcare Financing, Coverage and Access, Quality of Care, Healthcare Delivery, Regulatory Framework, Public Health Initiatives, Healthcare Workforce, Equity and Accessibility, Health Information Systems, and Emergency Preparedness to provide a general comparison framework. To structure the comparative analysis, 10 key domains of national MHSs' performance were considered, including financing, coverage, access, quality, delivery organization, regulation, public health, workforce, equity, and emergency preparedness [4, 24, 25, 26]. These domains are summarized in Table A1 in Appendix A, which provides concise definitions for each dimension and ensures consistency across countries.

Data can be displayed visually in tables, graphs, or diagrams for each aspect. Examples include bar graphs comparing healthcare expenditure as a percentage of GDP, pie charts displaying healthcare funding sources, line graphs showing life expectancy trends, and maps demonstrating healthcare access discrepancies by region. However, MHS change and data collection methods vary, making it difficult to gather comprehensive and up‐to‐date statistics for all countries. Data interpretation is also complicated by differences in population demographics, culture, and healthcare priorities. Interpretation must be cautious, as cross‐country comparisons are influenced by demographic, cultural, and systemic differences.

To this end, a comparative summary of selected national MHSs, presenting their key features, strengths, and weaknesses in a matrix format, can highlight broad patterns across financing models and governance structures [3, 27, 28, 29]. This is shown in Table A2 in Appendix A for Canada, France, Germany, Japan, the UK, and the USA.

**Table 2:** EU and other selected European countries physicians by specialty in 2021.

	(Number)								(per 100,000 inhabitants)							
	Total	General medical practitioners	General pediatricians	Gynecologists and obstetricians	Psychiatrists	Medical group of specialists	Surgical group of specialists	Other specialists not elsewhere classified	Total	General medical practitioners	General pediatricians	Gynecologists and obstetricians	Psychiatrists	Medical group of specialists	Surgical group of specialists	Other specialists not elsewhere classified
Belgium	41.611	14.041	1.647	1.590	2.024	10.805	7.469	—	356.3	120.2	14.1	13.6	17.3	92.5	64.0	—
Bulgaria	29.538	3.973	1.438	1.791	683	12.848	8.388	294	444.6	59.8	21.7	27.0	10.3	193.4	126.3	4.4
Czechia	46.051	8.428	1.670	3.123	1.785	17.447	10.709	222	431.5	79.0	15.7	29.3	16.7	163.5	100.4	2.1
Denmark (^1^)	26.344	4.694	538	701	1.137	4.822	3.910	226	449.8	80.2	9.2	12.0	19.4	82.3	66.8	3.9
Germany	381.249	88.286	15.381	21.783	23.796	125.227	104.587	2.189	455.0	105.4	18.4	26.0	28.4	149.4	124.8	2.6
Estonia	4.683	1.182	150	313	275	1.644	1.119	0	347.2	87.6	11.1	23.2	20.4	121.9	83.0	0.0
Ireland (^2^)	17.180	8.992	530	418	1.104	2.152	1.225	2.615	332.6	174.1	10.3	8.1	21.4	41.7	23.7	50.6
Greece	68.469	4.779	4.526	3.541	2.691	28.106	15.709	496	656.0	45.8	43.4	33.9	25.8	269.3	150.5	4.8
Spain (^3^)	205.366	45.460	13.504	6.189	6.233	57.773	50.763	836	430.0	95.2	28.3	13.0	13.1	121.0	106.3	1.8
France (^4^)	217.441	93.570	8.620	8.001	15.474	54.625	33.287	3.864	319.5	137.5	12.7	11.8	22.7	80.3	48.9	5.7
Croatia	15.272	3.190	1.008	888	715	6.145	3.297	29	396.0	82.7	26.1	23.0	18.5	159.3	85.5	0.8
Italy	249.869	47.253	16.242	12.996	12.019	97.237	64.122	0	423.4	80.1	27.5	22.0	20.4	164.8	108.7	0.0
Cyprus	4.735	1.257	303	219	127	1.521	1.308	0	518.8	137.7	33.2	24.0	13.9	166.7	143.3	0.0
Latvia	6.391	1.435	234	363	283	2.042	1.411	623	340.1	76.4	12.5	19.3	15.1	108.7	75.1	33.2
Lithuania	12.580	2.951	484	662	713	4.480	3.164	126	444.3	104.2	17.1	23.4	25.2	158.2	111.7	4.5
Luxembourg	—	—	—	—	—	—	—	—	—	—	—	—	—	—	—	—
Hungary	33.473	6.647	2.344	1.547	1.457	13.139	7.279	962	347.1	68.9	24.3	16.0	15.1	136.2	75.5	10.0
Malta	2.384	439	118	89	75	540	504	—	448.5	82.6	22.2	16.7	14.1	101.6	94.8	—
Netherlands	69.404	32.471	1.969	1.730	4.482	16.900	7.745	4.107	392.1	183.4	11.1	9.8	25.3	95.5	43.8	23.2
Austria	49.242	13.214	1.628	2.089	1.983	11.591	10.113	60	544.6	146.1	18.0	23.1	21.9	128.2	111.9	0.7
Poland	131.426	36.924	6.685	6.476	4.942	45.934	29.644	821	356.9	100.3	18.2	17.6	13.4	124.8	80.5	2.2
Portugal	59.695	31.673	2.327	1.966	1.565	14.004	8.527	891	573.5	304.3	22.4	18.9	15.0	134.5	81.9	8.6
Romania	69.487	15.140	3.120	3.088	3.077	30.181	14.460	421	364.8	79.5	16.4	16.2	16.2	158.4	75.9	2.2
Slovenia (^5^)	7.122	1.453	686	406	365	2.420	1.643	63	337.2	68.8	32.5	19.2	17.3	114.6	77.8	3.0
Slovakia	20.234	—	—	—	—	—	—	—	372.5	—	—	—	—	—	—	—
Finland (^1^)	15.818	6.862	386	461	610	2.187	1.988	3.324	285.5	123.8	7.0	8.3	11.0	39.5	35.9	60.0
Sweden (^1^)	45.916	6.478	1.111	1.445	2.286	10.624	7.180	916	440.8	62.2	10.7	13.9	22.0	102.0	68.9	8.8
Iceland (^1^)	1.631	211	19	60	75	391	285	0	437.8	56.6	5.1	16.1	20.1	105.0	76.5	0.0
Liechtenstein	143	51	4	6	17	32	33	0	362.1	129.1	10.1	15.2	43.1	81.0	83.6	0.0
Norway	26.783	5.507	969	690	1.295	4.782	3.138	479	490.8	100.9	17.8	12.6	23.7	87.6	57.5	8.8
Switzerland	39.362	10.094	2.121	2.011	4.662	7.461	7.711	192	448.5	115.0	24.2	22.9	53.1	85.0	87.9	2.2
Montenegro (^1^)	1.738	321	184	123	65	682	363	0	280.7	51.8	29.7	19.9	10.5	110.1	58.6	0.0
North Macedonia (^1^)	6.316	1.888	396	432	214	2.167	1.063	156	323.4	96.7	20.3	22.1	11.0	111.0	54.4	8.0
Serbia (^6^)	21.048	6.861	1.830	1.260	784	5.440	3.808	1.065	313.3	102.1	27.2	18.8	11.7	81.0	56.7	15.9
Türkiye	194.688	61.875	10.188	9.699	6.183	56.675	45.797	4.271	229.1	72.8	12.0	11.4	7.3	66.7	53.9	5.0

*Note:* (^1^): 2020. (^2^): Analysis by specialty only concerns physicians and hospitals. (^3^): Excludes stomatologists, dentists, interns, and residents. (^4^): Surgical group of specialists: definition differs. (^5^): Only includes physicians in institutions under the Ministry of Health. (^6^): Excludes the private health sector.

**Figure 1:**
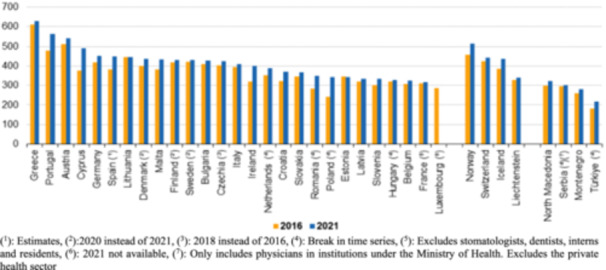
Practicing physicians, in 2016 and 2021 (per 100,000 inhabitants), per the EU and other selected European countries. (^1^): Estimates. (^2^):2020 instead of 2021. (^3^): 2018 instead of 2016. (^4^): Break in time series. (^5^): Excludes stomatologists, dentists, interns, and residents. (^6^): 2021 not available. (^7^): Only includes physicians in institutions under the Ministry of Health. Excludes the private health sector.

**Figure 2:**
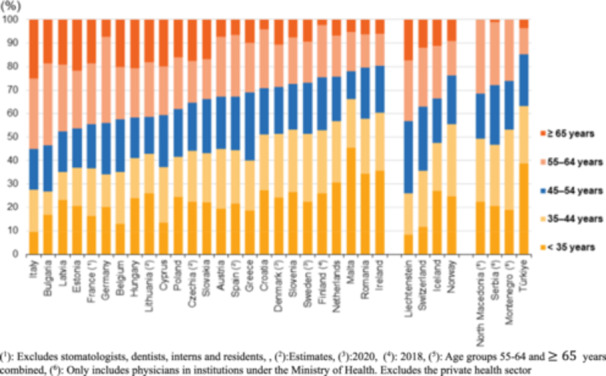
Percentage of physicians by age, in 2021, per the EU and other selected European countries. (^1^): Excludes stomatologists, dentists, interns, and residents. (^2^): Estimates. (^3^): 2020. (^4^): 2018. (^5^): Age groups 55–64 and ≥65 years combined. (^6^): Only includes physicians in institutions under the Ministry of Health. Excludes the private health sector.

To provide context on workforce capacity, the EU physician supply indicators between 2016 and 2021 were reviewed. Most member states reported growth in the number of medical graduates and practicing physicians per 100,000 population, though the magnitude of increase varied substantially across countries [8, 30, 31, 32]. As shown in Table 2 and Figures 1, 2 and 2, growth was uneven, with some countries experiencing rapid expansion in certain specialties, while others continued to face persistent shortages despite overall improvements. Detailed breakdowns by specialty, age, and country are presented below to illustrate this variation.

Figure 1 shows the practicing physicians, in 2016 and 2021 (per 100,000 inhabitants), while Figure 2 shows the % of physicians, by age, in 2021, per the EU and other selected European countries. Among the EU Member States, Greece had the highest number of physicians (in relation to population), at 629.2 per 100,000 inhabitants in 2021, a substantially higher number than any other EU member state. In 2021, around 469,000 generalist medical practitioners (GPs) were present throughout the EU, omitting statistics from Luxembourg, Slovakia, and Finland. France had the highest number of general practitioners (GPs) (94,000), followed by Germany (86,000); however, Portugal and Ireland reported the highest number of GPs per 100,000 population (298.2 and 233.3, respectively). In 2021, the largest percentages of physicians who were GPs were in Ireland (58%), Portugal (53%), and the Netherlands (47%). In 2021, more surgical experts than GPs were present in half of the EU member states for which data is accessible. The largest quantities of surgical experts were recorded in Germany (104,000) and Italy (62,000). Greece recorded the highest incidence rate per 100,000 population at 147.1, followed by Cyprus at 138.8, based on 2020 statistics. Malta was the sole member state to report a greater number of surgical specialists (512) than medical specialists (493) or GPs (440). The healthcare workforce in many of the EU is seeing a swift aging as the baby‐boomer generation approaches retirement age. In 2021, the proportion of physicians aged 55 years and older exceeded 40% in 10 EU Member States. The proportion exceeded 50% in Italy and Bulgaria, at 55.2% and 53.6%, respectively. In the majority of the remaining Member States with accessible data, the proportion of this age group among the total number of physicians ranged from 24.3% to 38.2%; lower percentages were seen in Malta (21.9%), Romania (20.4%), and Ireland (19.7%). The largest percentages of younger physicians (under 35 years) among those aged 55 and above were found in Malta (45.5%), Ireland (35.5%), and Romania (34.4%).

A comparison between 2016 and 2021 indicates that the number of medical doctors graduating per 100,000 population increased in almost all EU Member States, with the sole exception of Austria, which experienced a modest decline. The rise in most Member States may indicate a decline in population and/or an augmentation in the number of graduates. The most significant increases, in relative terms, were observed in Latvia, Bulgaria, Romania, and Cyprus.

Also, significant disparities exist among EU Member States concerning the proportion of each gender within the total physician population. In the past decade, there has been a gradual increase in the proportion of female physicians. Estonia and Latvia exhibited the highest proportions of female doctors, at 73% and 74%, respectively. The highest proportion of male physicians, at 61%, was observed in Cyprus. In Greece, Malta, and Italy, the proportions of male physicians were notably high, ranging from 55% to 57%. The minimal disparities between the shares of the two genders, each at 2 percentage points (pp), were recorded in Austria and Sweden according to 2020 data.

The second step was to provide facts regarding timelines and the actual implementation of the MHS of Cyprus, the actual subject of this study. Cyprus's General Health System (GHS) was officially implemented in 2019. Phase 1 (June 2019) introduced population‐wide outpatient care, while Phase 2 (June–December 2020) expanded to inpatient services and pharmaceuticals. The Health Insurance Organization (HIO) manages the National Health Insurance Fund and contracts with both public and private providers. This timeline is based on secondary sources summarizing the reform stages [18, 22, 23, 24, 33, 34] (see Appendix B).

As the goal here was to conduct a questionnaire for the evaluation of the Cyprus MHS, one could follow certain important factors related to surveys in MHS, namely, Assessment of Access, Quality of Care, Identifying Gaps, Health Outcomes, Policy Evaluation, Equity and Inclusivity, and Future Planning [13]. These are addressed in Table 3.

**Table 3:** Survey domains and rationale (aligned with comparison framework).

Aspect	Comment
Access to healthcare services	A well‐functioning medical health system (MHS) ensures that individuals can access necessary healthcare services when needed, regardless of factors such as income, location, or pre‐existing conditions. This access is essential for early detection and treatment of illnesses, preventive care, management of chronic conditions, and overall well‐being.
Healthcare quality	A strong MHS strives to provide high‐quality care that meets established standards and guidelines. This includes accurate diagnosis, effective treatment options, appropriate medication management, and adherence to patient safety protocols. Quality healthcare helps improve health outcomes and reduces the risk of complications or adverse events.
Disease prevention and health promotion	MHS play a crucial role in promoting public health and preventing the spread of diseases. This involves initiatives such as vaccination programs, screenings for common health conditions, health education campaigns, and efforts to address risk factors such as smoking, poor nutrition, and sedentary lifestyles.
Emergency and trauma care	During emergencies, accidents, or natural disasters, a well‐equipped MHS can provide timely emergency medical services, trauma care, and critical interventions. Access to emergency care can significantly reduce mortality rates and improve the chances of recovery for individuals facing life‐threatening situations.
Financial protection	Healthcare costs can be a significant burden for individuals and families, particularly in the event of serious illnesses or injuries. A comprehensive MHS, which may include health insurance coverage or government‐funded healthcare programs, helps provide financial protection by reducing out‐of‐pocket expenses and ensuring that individuals can afford necessary medical care without facing financial hardship.
Health equity and social justice	A fair and equitable MHS strives to address disparities in healthcare access and outcomes among different population groups. This includes efforts to reduce barriers to care for marginalized communities, improve health literacy, and address social determinants of health such as poverty, discrimination, and inadequate access to education and resources.
Economic development	A healthy population is essential for economic prosperity. A MHS that effectively promotes health and provides timely healthcare services can contribute to higher productivity, reduced absenteeism, and lower healthcare‐related costs for businesses. It also fosters innovation and entrepreneurship in the healthcare sector, driving economic growth and job creation.

*Note:* Domains were adapted for questionnaire design to capture citizen perceptions of financing, access, quality, and reliability.

Based on literature and the steps above, a questionnaire was created to assess MHS and their features, including factors Satisfaction, Trust, Reliability, Expectations, Improvement factors, and HS Comparison before and after the GHS was implemented. Each variable was represented by categorized Likert‐scale questions in the questionnaire. The questions were chosen based on theoretical foundations, a literature research, and qualitative interviews with the authors' peers. Responses were anticipated to help evaluate system efficacy and identify areas for improvement. Surveys are essential for assessing a national healthcare system and promoting access, quality, and equity. Questionnaire development included expert consultation with health‐policy academics and survey‐methods specialists. Cognitive pretesting with a small group (*n* ≈ 10–15) ensured clarity, followed by a pilot survey (*n* ≈ 40–60) to refine timing and layout. Content validity was checked against established domains, aiming for CVI ≥ 0.80. The six constructs (Satisfaction, Trust, Reliability, Communication, Experience, and Importance) were prespecified, and internal consistency was later assessed with Cronbach's *α*.

Following a pilot survey in all of Cyprus, the final version of the questionnaire consisted of the following sections: (i) Demographic information about the respondent, which included gender, geographical district, age group, annual income, work sector; (ii) Likert scale (1–5) questions covering the six factors mentioned above. The Likert scale decoding per factor can be found in Table 4, while all demographic characteristics of the sample can be found in Table 5.

**Table 4:** Likert scale decoding.

Factor	Likert scale (1–5)				
	1	2	3	4	5
Satisfaction	Very Dissatisfied	Dissatisfied	Neutral	Satisfied	Very Satisfied
Trust	No Trust	Low Trust	Moderate Trust	High Trust	Complete Trust
Reliability	Very unreliable	Unreliable	Neutral	Reliable	Very reliable
Comparison	Very Negative Impact	Negative Impact	No significant Impact	Positive Impact	Very Positive Impact
Expectations	Very low	Low	No Expectations	High	Very high
Improvement factors	Strongly Disagree	Disagree	Neutral	Agree	Strongly Agree

**Table 5:** Demographic sample characteristics.^a^

Gender	Sample size	Sample (S) %	Population (P) %	District residence	Sample size	S %	P %	Age group	Sample size	S %	P %
Men	221	49.7	48.5	Limassol	148	33.26	32.00	16–18	26	5.84	4.00
Women	224	50.3	51.5	Nicosia	136	30.56	34.00	19–34	95	21.35	21.20
				Larnaca	67	15.06	17.00	35–49	117	26.29	23.00
				Paphos	55	12.36	11.00	50–64	109	24.49	18.80
				Famagusta	39	8.76	6.00	65–	98	22.02	16.70

**Annual income** (€)		**Sample size**	**S %**	**Work sector**		**S size**	**S %**	**Citizenship**		**S size**	**S %**
0–10,000		41	9.2	Medical		71	16.0	Cypriot		364	81.2
10,001–20,000		48	10.8	Educational		96	21.6	Greek		47	10.6
20,001–30,000		71	16.0	Business		89	20.0	Rest of Europe		14	3.1
30,001–40,000		182	40.9	Services		122	27.4	Asia		10	2.2
40,001–		103	23.1	Construction		67	15.1	America		10	2.2

^a^ The participants were selected according to the official demographic distributions provided by the Statistical Service of Cyprus.

Skilled enumerators collected data via oral or telephone interviews using tablets or by having respondents complete a questionnaire. All the enumerators held at least undergraduate qualifications in social sciences or related fields. Before data collection, they underwent targeted training sessions on interview techniques, questionnaire administration, and confidentiality procedures, ensuring standardized and reliable data collection across all districts. The law requires the Statistical Service to keep all acquired data confidential and use it only for statistical purposes [34]. A stratified sampling (see Table 5) was used to identify 445 eligible Cyprus GHS. The questionnaire's responses with regard to the individual factors undergo a descriptive analysis, as well as a reliability analysis in Section Conclusion, 4.

## 3 Statistical Analysis Results

Descriptive statistics (means and standard deviations) were calculated for all items and constructs. The data obtained from the designed questionnaire were inserted into an MS Excel worksheet and subsequently into SPSS, where all analyses were performed. Recall that (Section Methods, 2), upon construction of the questionnaire, questions grouped into six constructs: Satisfaction (SAT), Trust (TRU), Reliability (REL), Comparison (COM), Expectations (EXP), and Improvement Factors (IMP), were posed to the survey participants, using a Likert scale from 1 to 5. These six factors were selected as they reflect core dimensions of MHS evaluation highlighted in prior studies [4, 25, 26, 27] and align with the comparison framework used in this analysis.

Before moving to the detailed results obtained per item and factor, a reliability analysis was performed on the factors. Construct reliability for each factor was tested through calculating Cronbach's alpha (*α*) values (along with 95% confidence intervals); it turned out that all factors had very good or excellent construct reliability with *α* over 0.80 [35]; namely, 0.92 (0.88, 0.95) for SAT, 0.93 (0.89, 0.96) for TRU, 0.90 (0.86, 0.94) for REL, 0.88 (0.84, 0.92) for COM, 0.89 (0.85, 0.92) for EXP, and 0.80 (0.75, 0.85) for IMP, respectively.

Then the descriptive statistics (mean per Likert score, but also mean and standard deviation [SD] per item [question]) are summarized in Tables 6, 7, 8 for each factor.

**Table 6:** Satisfaction, trust, and reliability with the GHS.

SAT	Percentage (%)											Likert 1–5				
How satisfied are you with GHS for the:	1		2		3	4			5			Mean				SD
1. Access to healthcare services (availability of doctors, specialists, hospitals)	1.80		23.15		58.43	13.71			2.92			2.928				0.743
2. Quality of healthcare services received	4.04		65.39		17.53	10.56			2.47			2.420				0.827
3. Waiting times for appointments or treatments	13.26		67.64		13.71	3.82			1.57			2.128				0.740
4. Effectiveness of preventive care services (screenings, vaccinations)	1.57		12.36		72.81	7.19			6.07			3.038				0.707
5. Professionalism and competence of healthcare professionals	1.57		61.57		24.27	10.11			2.47			2.503				0.795
6. Information and communication provided regarding your healthcare needs and options	1.35		60.22		21.12	12.58			3.82			2.966				0.593
7. The cost‐effectiveness of the healthcare services	2.25		60.22		21.12	12.58			3.82			2.555				0.879
8. Personalized and patient‐centered care	4.04		69.89		13.03	10.56			2.47			2.375				0.822
9. Overall satisfaction with the National Health System	6.74		62.25		18.43	11.01			1.57			2.384				0.828
Average percentages per Likert score	4.07		48.39		34.98	9.71			2.85			2.589				0.600

**TRU**	**Percentage (%)**													Likert 1–5		
How much do you trust GHS:	1		2	3				4			5			Mean		SD
1. To meet your healthcare needs effectively	1.80		66.52	20.67				10.34			0.67			2.416		0.725
2. To ensure the privacy and security of your personal health information	1.35		51.46	32.36				12.13			2.70			2.634		0.815
3. To have your best interests in mind	4.72		64.94	22.02				7.64			0.67			2.346		0.717
4. To employ professionals who are competent and knowledgeable	5.17		64.72	21.57				6.97			1.57			2.351		0.751
5. To provide timely and accurate information about your health	1.80		64.42	17.75				11.91			1.12			2.431		0.766
6. To make evidence‐based decisions for the overall benefit of the population	6.07		69.44	17.30				6.97			0.22			2.258		0.682
Average percentages per Likert score	3.49		64.08	26.95				9.33			1.16			2.406		0.641

**REL**	**Percentage (%)**												Likert 1–5			
How Reliable is the GHS:	1	2		3			4			5			Mean		SD	
1. In providing timely access to healthcare services when needed	6.52	60.22		24.04			8.76			0.45			2.364		0.751	
2. In delivering accurate diagnoses and effective treatments	31.24	43.15		11.91			13.03			0.67			2.088		1.000	
3. In handling emergency situations efficiently	2.92	63.37		22.70			10.79			0.22			2.420		0.729	
4. In the competence and professionalism of healthcare providers	4.72	62.62		23.15			5.62			0.90			2.324		0.692	
5. In effectively addressing public health emergencies and crises	1.57	63.60		24.27			10.11			0.45			2.443		0.712	
Average percentages per Likert score	9.39	59.19		21.21			18.16			0.54			2.328		0.668	

**Table 7:** Comparison before and after the implementation of the GHS and expectations from the GHS.

COM	Percentage (%)					Likert 1–5	
	1	2	3	4	5	Mean	SD
1. The impact of the GHS on your ability to access necessary healthcare services	2.02	15.96	72.81	7.42	1.80	2.910	0.615
2. The impact of the GHS on your overall well‐being and quality of life	0.90	17.98	67.87	12.58	0.67	2.942	0.604
3. Improvements in the accessibility of healthcare	1.35	61.35	25.84	10.11	1.35	2.488	0.748
4. The quality of healthcare services and facilities	1.80	11.69	76.85	7.64	2.02	2.964	0.587
5. The affordability of healthcare services	0.90	6.97	73.71	13.93	4.49	3.142	0.636
6. The waiting times for medical appointments	8.54	26.07	58.88	5.62	0.90	2.643	0.753
7. The coordination and communication among healthcare providers	2.02	63.15	27.42	6.07	1.35	2.416	0.697
8. The healthcare costs are more transparent and understandable	2.25	68.54	14.83	11.24	3.15	2.445	0.840
9. Did you receive more personalized and patient‐centered care?	3.82	69.66	16.63	8.54	1.54	2.339	0.743
The integration of technology in healthcare services	4.49	12.58	68.76	10.79	3.37	2.960	0.739
Average percentages per Likert score	2.81	35.40	50.36	9.39	2.04	2.725	0.482

**EXP**	**Percentage (%)**					Likert 1–5		
	1	2	3	4	5	Mean	SD	
1. The primary goal of GHS is to provide universal access to healthcare services	0.22	6.74	6.97	78.65	7.42	3.863	0.644	
2. The primary goal of GHS is to control healthcare costs	1.12	5.62	11.01	76.18	6.07	3.804	0.677	
3. The role of the government in GHS is to regulate and oversee healthcare providers	4.72	3.15	7.42	75.28	9.44	3.816	0.828	
4. The role of the government in GHS is to fund healthcare services through taxes	4.72	2.47	9.66	24.72	58.43	4.297	1.057	
5. The GHS should cover primary care	0.45	3.60	5.39	69.44	21.12	4.072	0.667	
6. The GHS should cover emergency care	0.22	0.90	4.94	22.70	71.24	4.638	0.641	
7. The GHS should cover preventive care (vaccinations, screenings)	0.45	0.67	5.84	65.39	27.64	4.191	0.605	
8. The GHS should cover specialty care (e.g., cardiology, orthopedics)	0.22	1.12	5.17	19.10	74.38	4.663	0.649	
9. The GHS should cover mental health services	0.22	1.12	6.07	64.72	27.87	4.189	0.607	
The GHS should cover dental and vision care	0.22	1.35	4.94	19.10	74.38	4.661	0.656	
The monthly cost of GHS is a major concern	0.45	1.35	8.54	14.83	74.83	4.622	0.735	
Average percentages per Likert score	1.18	2.55	6.90	48.19	41.18	4.256	0.497	

**Table 8:** GHS improvement factors.

	Percentage (%)					Likert 1–5	
	1	2	3	4	5	Mean	SD
1. Individuals should have the option to purchase private health insurance in addition to the GHS	1.80	3.15	8.09	14.30	72.50	4.528	0.903
2. Individuals should have the option to choose between private health insurance and GHS	1.35	2.25	7.64	13.90	74.80	4.587	0.832
3. The access to healthcare Services is sufficient (primary care, specialized medical services)	2.47	18.40	64.0	12.10	2.92	2.946	0.720
4. The monthly cost (contribution rate based on income) for individuals is high	3.60	48.90	11.90	15.73	19.78	2.991	1.258
5. The quality of care is high (timely and appropriate treatment, healthcare professionals are well‐trained and skilled)	3.37	71.01	11.69	11.91	2.02	2.382	0.814
6. The level of health education and promotion is high (initiatives to promote healthy lifestyles, effective awareness programs, prevention, and early intervention strategies)	4.94	22.02	59.10	12.36	1.57	2.836	0.760
7. The level of healthcare infrastructure is high (modern and up‐to‐date, facilities are well‐maintained and adequately equipped, sufficient number of healthcare facilities)	1.80	22.70	62.25	12.13	1.12	2.881	0.672
8. The level of the healthcare workforce is high (adequate number of healthcare professionals, healthcare workforce is well‐distributed across regions, programs to continuously train and develop healthcare professionals)	6.52	69.89	14.38	7.87	1.35	2.276	0.754
9. The level of the healthcare financing is high (affordability of healthcare services)	4.72	11.69	69.44	13.03	1.12	2.942	0.691
The level of health information system is high (health records and information are securely managed and protected)	0.67	65.17	23.60	8.31	2.25	2.463	0.750
The level of the patient satisfaction and feedback is high (there are channels for patients to provide feedback and suggestions, and feedback is actively used for improving services)	5.84	17.98	67.87	6.74	1.57	2.802	0.710
The level of government policies and regulations is high (effective regulation of healthcare providers and services, policies are flexible and adaptive to changing healthcare needs)	1.80	72.13	14.38	10.34	1.35	2.373	0.747
The level of emergency response and preparedness is high (well‐prepared for emergencies, response times during emergencies are satisfactory, emergency services are well‐coordinated)	6.07	70.56	11.01	10.79	1.57	2.312	0.804
Average percentages per Likert score	3.46	45.14	32.72	11.51	7.17	2.948	0.444

The overall Satisfaction, Trust, and Reliability scores are moderately low (means < 3 on the 5‐point Likert scale), indicating a need for further analysis to identify underlying causes.

The mean value for all (but one) items and the average of factor COM in Table 7 are less than 3 indicating that the overall comparison before and after the implementation of GHS is moderately negative. The only item that has a slightly positive impact is the affordability of healthcare services. The mean value for all items and the average of factor EXP in Table 9 are over 3 indicating that the people are expecting more from the GHS in all aspects, verifying in a way the results for factors SAT, TRU, REL, and COM above.

**Table 9:** Mean values for each construct.

	SAT	TRU	REL	COM	EXP	IMP
Gender						
Male	2.64	2.47	2.42	2.76	4.24	2.99
Female	2.54	2.35	2.24	2.69	4.28	2.91
Age						
16–18	2.99	2.85	2.72	2.92	3.96	2.96
19–34	2.47	2.31	2.15	2.68	4.27	2.89
35–49	2.59	2.43	2.30	2.76	4.26	2.90
50–64	2.51	2.32	2.26	2.65	4.17	2.92
65–	2.68	2.45	2.51	2.75	4.41	3.09
Education						
Primary school	3.71	3.70	3.84	3.52	4.56	3.82
High school	2.84	2.73	2.67	2.90	4.21	3.10
University	2.49	2.28	2.19	2.66	4.26	2.89
Annual income (€)						
0–10,000	3.01	2.86	2.80	2.93	4.01	3.02
10,001–20,000	3.30	3.03	3.10	3.15	4.36	3.34
20,001–30,000	2.62	2.54	2.48	2.80	4.23	3.03
30,001–40,000	2.40	2.21	2.10	2.61	4.29	2.86
40,001–	2.41	2.19	2.07	2.60	4.27	2.84
Work sector						
Medical/Health	2.52	2.37	2.30	2.62	4.16	2.87
Educational	2.62	2.45	2.38	2.77	4.18	2.95
Business	2.48	2.28	2.17	2.67	4.29	2.85
Services	2.60	2.40	2.34	2.76	4.37	3.03
Other	2.73	2.55	2.48	2.77	4.21	3.00
District residence						
Limassol	2.69	2.55	2.48	2.77	4.15	2.98
Nicosia	2.54	2.34	2.29	2.68	4.32	2.90
Larnaca	2.61	2.44	2.32	2.77	4.25	3.02
Paphos	2.44	2.22	2.13	2.68	4.38	2.92
Famagusta	2.55	2.30	2.18	2.71	4.28	2.93

The mean value for all (but two) items and the average of IMP in Table 8 are less than 3, indicating the need for a lot of improvements of the existing health system (the GHS). It is worth mentioning that overall individuals believe that they should have the option to purchase private health insurance in addition to the GHS and/or should have the option to choose between private health insurance and the GHS (the first two items that are the only with average values well over 4). This is a consequence resulting from the controversy over payer type (single or multi‐payer) that surfaced among political and social discussions in Cyprus during the preparation phase of the GHS.

Finally, a very interesting point of investigation was whether or not the results of all the responses of the 445 participants had noticeable variation according to gender, age group, education level, annual income, working sector, and district of residence. A detailed descriptive analysis for investigating that was performed, as shown in Table 9.

## 4 Discussion

The main conclusions that emerge from the statistical analysis performed on all questionnaire factors under investigation are summarized as follows.

For all aspects that criticize the GHS, such as SAT, TRU, REL, COM, and IMP, respondents score below average (on a Likert scale from 1 to 5 with a theoretical mean of 3), indicating either skepticism or higher expectations. For the factor of favorable GHS criticism, EXP, respondents scored above average, confirming the aforementioned finding.

For the factor SAT, gender and age groups' responses are similar, except for age 16–18, which seems neutral. SAT exhibits a higher mean for primary school graduates and those with annual incomes under €20,000. Work Sector and District Residence groups' responses are similar for this construct.

For REL, there is no notable difference among the gender and age groups, except for age 16–18, which seems to have a neutral opinion.

As for COMP, again, no gender or age differences are observed, except for age 16–18, which appears neutral. For people with primary education and €10,001–20,000 income, COMP exhibits higher means. There is no notable difference between the Work Sector and District Residence responses for this construct.

For EXP, there is no notable difference among the gender and age groups, except—again—1 for age 16–18 having lower mean. Those with Primary Education have a higher. There is no notable difference between the Work Sector and District Residence for this construct.

For IMP, there is no notable difference among the gender and age groups, except for age 65+, which seems to have a slightly higher mean. Those with Primary Education €10,001–20,000 income have a higher mean. Again, there is no notable difference between the Work Sector and District Residence for this construct.

It is clear that inferential statistical analyses could be reported to formally obtain deeper insight into the reported findings above. These are left for a detailed future study, and could include: (i) ANOVA or Kruskal–Wallis tests to determine whether differences in mean scores across demographic groups (Table 9) are statistically significant; (ii) multiple linear regression to identify which demographic or system‐related factors (e.g., income, education, district) significantly predict Satisfaction, Trust, or Reliability.

Now, one needs to focus on the low average scores for SAT, TRU, and REL (2.589, 2.406, 2.328). Based on past surveys, this is expected. In 2001, a comparison of MHS in five countries—the USA, the UK, Canada, Australia, and New Zealand—found that 18%–25% (depending on the country) of respondents required only minor changes, while the rest required fundamental changes or complete restructure [16].

In 2023, a survey among European residents and health professionals yielded a mean Satisfaction of 56.5% and individual averages by nation between 35.3% and 73.4%, with 9 out of 27 countries, including Cyprus, below 50% [36]. In 2010, 17.4% to 56.4% (depending on country) of nine former Soviet Union countries were at least quite satisfied, slightly up from 2001 [37]. The 2011–2013 International Social Study Program health care study in 31 countries found low mean Trust values (below 50%) for 21 nations [38].

MHS Satisfaction in Europe varies greatly [39]. With low out‐of‐pocket costs and strong primary care networks, the Netherlands (46%), Germany (39%), and the UK (53%) have some of the happiest populations. These countries have economic healthcare, reducing financial barriers to accessing essential services and increasing satisfaction. With comprehensive coverage policies that guarantee after‐hours healthcare, the Netherlands and Germany rank high in availability and affordability. Poland (13%), Romania (11%), and other ex‐Eastern European countries express lesser satisfaction. Residents in these nations struggle to get timely care due to healthcare access, availability, and financing [39]. Thus, satisfaction ratings in these countries are generally low. Countries with strong public investment and coverage, like ex‐Western Europe, have greater satisfaction rates than nations with lower healthcare resources or limited access for particular demographic segments.

A 2023 Eurobarometer survey found that only 52% of Europeans like their MHS, with notable variations among countries. Germany has a 75% Satisfaction rate, while Bulgaria and Romania have 30%, reflecting budget and healthcare infrastructure differences. Countries like the Netherlands, Denmark, and Finland rate higher on Satisfaction and service accessibility, whereas others, especially ex‐Eastern Europe, are struggling with health spending, workforce development, and mental health services [14, 15].

## 5 Conclusions

The General Healthcare System (GHS) of Cyprus has been operating for 5 years. It may be Cyprus's greatest social success in recent times. Over the past 5 years, most medical centers in Cyprus and most specialized doctors have contracted with the GHS, allowing citizens to get medical services from a wide selection of specialists and medical centers. Only 6 of the 78 medically licensed Medical Centers are not GHS‐contracted. This GHS evaluation can help improve flaws and preserve and improve strengths. Due to its variety and affordability, the GHS gives all citizens easier access to most medical services, especially the low‐income population, who until 2019 could only use public hospitals and not private ones, which were only available to the wealthy.

However, this almost free provision of services has also created some distortions that definitely need analysis and improvement [40, 41, 42]. According to Health Systems and Policy Monitor (HSPM) [40], increased visitation to medical services has created long wait lists for serious medical cases and specialized care. As a result, urgent cases cannot be served in a timely and proper manner, creating annoyance and frustration with the GHS operation. Generally, as seen from Table 1, people do not show satisfaction with the GHS, while one would expect that with the establishment of this new MHS and in relation to the previous situation of congestion in the limited State Hospitals of the majority of citizens, the level of satisfaction and trust would be much higher. It is also evident that patients who had private health insurance before the establishment of the GHS are now worse off, with lower‐quality healthcare. In well‐designed universal health systems, private insurance is not intended to duplicate the statutory benefits; rather, it typically covers cost‐sharing or noncore services, and its market often shrinks (“crowd‐out”) when universal entitlements expand [43]. Hence, requiring people (Cypriots in this case) to finance both a full statutory package and a parallel full private package is therefore atypical and raises efficiency and equity concerns. This is evident in Table 8, where citizens believe they should be able to choose between private insurance and GHS contributions, with one complementing the other without forcing double or triple medical costs. Since GHS contributions are based on each citizen and private company's total profits (pay and other earnings), the cost of accessing the same medical treatments is disproportionate based on economic criteria. This, according to Table 9, seems to benefit low‐wage workers over higher‐wage ones.

According to the OECD [41], the introduction of the GHS significantly expanded citizens' access to primary care, specialized services, and emergency care, providing a broader range of medical options than before its implementation. However, both the WHO and HSPM [40] highlight that the GHS has faced challenges related to increased and sometimes unjustified demand for services, alongside insufficient regulatory oversight, which has led to inefficiencies in resource allocation and service delivery. Recent reviews further indicate that a considerable number of foreign‐trained physicians entered the Cypriot system without sufficient orientation or integration measures, creating disparities in clinical standards and patient trust [42]. Combined with high daily patient volumes, this situation often limits the time available for comprehensive medical examinations, potentially affecting both diagnostic quality and patient satisfaction [41]. At the same time, GHS has made healthcare financially accessible to a much broader share of the population; however, this increased accessibility has also resulted in overutilization of services, constraining efforts to control healthcare expenditures. On a positive note, the 5‐year review by the European Observatory shows that most beneficiaries continue to support the GHS's mission and remain optimistic about its future improvements, particularly regarding the system's sustainability and support for vulnerable populations. While systemic challenges persist, these findings suggest that public confidence in the GHS remains resilient, offering a strong basis for continued reform and optimization [40, 41, 42].

## Author Contributions

**George Evripides:** writing – original draft, software, methodology, investigation, formal analysis, conceptualization. **Paul Christodoulides:** writing – review and editing, writing – original draft, supervision, software, methodology, investigation, formal analysis, conceptualization.

## Conflicts of Interest

The authors declare no conflicts of interest.

## Transparency Statement

Paul Christodoulides affirms that this manuscript is an honest, accurate, and transparent account of the study being reported; that no important aspects of the study have been omitted; and that any discrepancies from the study as planned (and, if relevant, registered) have been explained.

## Data Availability

Data are available on request from the authors.

## Appendix A General Information About MHSs

**Table A1:** Key aspects used for comparison of MHS.

Category	Comparative aspect
Healthcare financing	− Compare the percentage of GDP spent on healthcare. − Breakdown of funding sources (government, private insurance, out‐of‐pocket payments). − Compare healthcare expenditure per capita.
Coverage and access	− Percentage of population covered by health insurance. − Availability of healthcare services (number of hospitals, clinics, doctors per capita). − Wait times for elective procedures.
Quality of care	− Healthcare outcomes (life expectancy, infant mortality rate, disease‐specific mortality rates). − Patient satisfaction ratings.
Healthcare delivery	− Compare primary care utilization. − Specialist availability and referral processes. − Telemedicine adoption rates.
Regulatory framework	− Compare healthcare regulations (licensing, accreditation, drug approval). − Malpractice and liability laws.
Public health initiatives	− Compare vaccination rates. − Disease surveillance and response capabilities. − Public health education and prevention programs.
Healthcare workforce	− Number of healthcare professionals per capita. − Nurse‐to‐patient ratios. − Physician satisfaction and burnout rates.
Equity and accessibility	− Disparities in healthcare access based on socioeconomic status, ethnicity, or geographic location. − Measures taken to address healthcare inequalities.
Health information systems	− Compare electronic health record adoption rates. − Interoperability of health information systems.
Emergency preparedness	− Availability of emergency medical services. − Disaster response capabilities.

**Table A2:** Strengths and weaknesses of several national MHS.^a^

Country	Features	Main strengths	Main weaknesses
Canada (Medicare)	The healthcare system of Canada provides universal coverage for medically necessary physician and hospital and services, and is publicly funded through taxation.	Universal coverage, emphasis on preventive care, and relatively low administrative costs.	Long wait times for elective surgeries and specialist consultations, challenges in funding pharmaceuticals and mental health services.
France	France has a mixed healthcare system with both public and private providers. It operates a universal health insurance system that covers a significant portion of healthcare costs.	High‐quality healthcare services, low out‐of‐pocket costs for patients, emphasis on preventive care, and health promotion.	Regional variations in healthcare quality and access, challenges in controlling healthcare spending.
Germany	Germany operates a social health insurance system, with citizens required to have health insurance, either statutory (SHI) or private (PHI).	High‐quality healthcare services, relatively short wait times, emphasis on patient choice and autonomy.	Rising healthcare costs, disparities in access between SHI and PHI beneficiaries, and challenges in integrating care across different providers.
Japan	Japan has a mixed healthcare system with universal coverage provided by a combination of employer‐based health insurance and government insurance for the elderly and unemployed.	Longevity and good health outcomes, relatively low healthcare costs, emphasis on preventive care, and primary care.	Aging population leading to financial sustainability concerns, and overuse of hospitals leading to overcrowding.
United Kingdom (NHS)	The NHS is publicly funded and provides comprehensive healthcare services, including primary, secondary, and tertiary care, funded primarily through taxation.	Universal coverage ensures access to healthcare for all residents, irrespective of ability to pay. Emphasis on equity and affordability is given.	Long wait times for nonemergency treatments, underfunding leading to staff shortages and infrastructure challenges.
United States of America	The USA healthcare system is primarily based on private health insurance, with some government‐funded programs like Medicare and Medicaid for certain populations.	Advanced medical technology, innovation, and high‐quality specialist care.	High healthcare costs, millions uninsured or underinsured, significant administrative burden, and disparities in access to care based on income, race, and geography.

a These are simplified summaries, and each country's MHS has nuances and complexities beyond what is outlined here. Additionally, ongoing reforms and changes in healthcare policies can impact the strengths and weaknesses of these systems over time.

## Appendix B The MHS of Cyprus

**Table B1:** Timeline of reform stages of GHS.

Year	Reform stage of GHS
2001	Legislation of the GHS was accordingly passed
2003	The health insurance organization was established
2014	Attempts for a multipayer system were resisted
2015	A study by the WHO (World Health Organization) concluded that only a single‐payer system is feasible in Cyprus
2017	The law for the state health services organization was approved by the House of Parliament
2017	The amended law of GHS was adopted unanimously by the House of Parliament
2019	The first stage of GHS was launched
2020	The second stage of GHS was launched

**Table B2:** Financing the GHS.

Contributions	Initial stage^a^ salary %	Final stage^b^ salary %
Employees	1.70	2.65
Employers	1.85	2.90
Self‐Employed	2.55	4.00
State	1.85	2.90
Pensioners	1.70	2.65
Income earners (rent, interest, dividends)	1.70	2.65
Public Officers	1.70	2.65
Government	1.65	4.70

**Copayment**		
Pharmaceuticals	1€ per product	
Per Laboratory tests	1€ per test or test panel	
Specialist Visit	6€ per visit	
Visit to a nurse or midwife	6€ per visit	
Healthcare service performed by a specialist doctor in radiology/diagnostic radiology	10€ per visit	
Allied healthcare provider	10€ per visit	
Emergency services	10€ per visit	
Visit to a specialist without referral from a Personal doctor	25€ per visit	

^a^ March 1, 2019 to May 29, 2020.

^b^ Full Implementation as of June 1, 2020.

**B.1 Timeline of reform stages of GHS**

Cyprus, along with Ireland, was the last EU country without a universal national MHS. Specifically, until 2019, Cyprus's healthcare landscape comprised two fragmented health sectors that operated concurrently and uncoordinatedly: the for‐profit private sector and the severely regulated public sector. Access to the (nearly free) public sector system was offered to anybody who met one of various socioeconomic or work status requirements. Individuals who did not match these criteria could only get privately funded, out‐of‐pocket (OOP) healthcare services from either the private or public sectors. Long waiting lists in the public sector, along with limited operation hours, made the private sector the only viable alternative for many patients, explaining the poor perceived added value of being a public healthcare system beneficiary. The healthcare services ecosystem resulted in large OOP payments. OOP payments were the primary source of healthcare spending (57% vs. 43% governmental funding) in 2018. During the same year, Cyprus' total health expenditure (THE) as a proportion of GDP was 6.7%, ranking among the lowest in Europe. Furthermore, the healthcare sector's horizontal segmentation slowed the implementation of supply‐ and demand‐side control measures such as clinical and therapeutic guidelines, co‐payment, medical audit programs, and pricing regulating mechanisms. Furthermore, a lack of cooperation between the two sectors resulted in very inefficient capacity planning in terms of both human resources and infrastructure at both ends of the spectrum, such as a glut of imaging centers and a scarcity of specific specialties, such as general practitioners. Public participation in strategic planning, prioritization, and resource allocation was extremely limited. The issue worsened following the 2013 financial crisis, which caused many healthcare professionals to leave the public sector due to pay cuts and uncertainty about their employment status as tenured public officials. This was exacerbated by the memorandum's requirement to freeze public sector recruitment until 2016, which increased wait times and put more strain on already overburdened resources. On an organizational level, the healthcare landscape in Cyprus includes three major public stakeholders. In 2017, as part of another memorandum‐imposed structural adjustment, the Cyprus Ministry of Health transferred operation of public hospitals to a newly formed organization, the State Health Services Organization (SHSO). The purpose of this reform is for the Ministry of Health (MoH) to focus on its primary regulatory duty while abandoning the other two competing responsibilities of payer and provider. SHSO enrolled in the GHS as a healthcare provider, much like the private ones. HIO, allocated the position of payer, was founded in 2001 as a public legal organization, and it is overseen by a Board of Directors who represent businesses, trade unions, patients, and the government. Its principal objective is to properly handle the GHS fund, contract with all healthcare providers, enroll beneficiaries, and assure patients' access to high‐quality healthcare services. In this atmosphere, and after years of simmering talks among social stakeholders that nearly erupted into civil war and political regressions, a single‐payer health insurance system was implemented on June 1, 2019. The GHS is based on a statute passed by Parliament in 2001 and modified in 2017. The procrastination was mostly due to concerns over the system's financial viability. Table B1 depicts the timing of the GHS reform steps in Cyprus.

**B.2 GHS inclusion of eligible beneficiaries**

The Fund's main beginning of payment is offerings levied on hires, incomes and pensions, and state revenues. Some of the applicable contributions are the following:

- Employees provide 2.65% on their wages.
- Employers contribute 2.90% on member earnings.
- Self‐employed provide 4.00% on their earnings.

The Government contributes 4.70% of member and independent gains. To ensure the GHS's long‐term commercial viability, its budget is managed in conjunction with a hard‐cap global budget. This compensation method allocates a substitute funding to various therapeutic efforts. Each healing exercise within a substitution budget is awarded a profit denoted in “parts.” The value of the “unit” is determined for each temporal period of an event or entity's existence and is a function of the total substitute‐budget amount assigned and the total number of “parts” used within that substitute‐budget. Thus, each time, the amount repaid for each activity varies, establishing the book acted. This allows physicians to counteract supplier‐inferred demand practices while also advancing chemists' ability to operate only the necessary number of medications. For pharmaceuticals, the total budget is achieved through claw‐backs that will be implemented before real strength expenditure exceeds the predetermined budget. Furthermore, physicians cannot offer to perform something for private subjects unless these services are clearly not below the GHS, for fear of establishing a parallel private market and potentially shifting beneficiaries to this health management road. Table B2 shows all contributions and relevant copayments for the financing of the GHS.

Until 2019, the healthcare environment in Cyprus comprised two disjointed sectors operating in parallel and without coordination: the profit‐driven commercial sector and the severely regulated state sector. Access to the predominantly complimentary public sector system was permitted to individuals who met one of various socioeconomic or employment status criteria. Individuals who did not satisfy these criteria could only get privately funded, out‐of‐pocket (OOP) healthcare services from either the private or public sector. Before the establishment of the GHS, socioeconomic and employment criteria governed membership in public risk‐sharing schemes. Before the establishment of the GHS, non‐EU nationals were required to provide proof of private health insurance to obtain a residency permit. There were specifically developed health insurance coverage schemes available commercially. These were fundamental insurance plans with minimal maximum reimbursement rates, frequently far lower than the real costs imposed by both public and commercial providers. Also, before the establishment of the GHS, refugees and their dependents, as well as individuals having supplemental protection status who were ordinary residents of the territories governed by the Republic of Cyprus, had access to complementary treatment, contingent upon the same requirements as Cypriot citizens. Enrollment was contingent upon a 3‐year uninterrupted subscription to the social insurance fund, which obstructed access.

After much deliberation, albeit with an extraordinary social cohesion and acceptance that preceded the GHS implementation, the GHS was finally implemented in 2019. The GHS implemented a set of efficiency measures. Major challenges, already encountered, include hospital enrollment and coordination between the public and private sectors. The implementation of the GHS represents a key change in the history of the Republic of Cyprus. The enduring fragmentation between public and private healthcare sectors, the highest OOP healthcare expenditures in Europe, and protracted waiting lists have necessitated the establishment of a contemporary, patient‐centered, efficient, and effective healthcare system.
